# PANoptosis nexus in ischemia-reperfusion injury: from integrated cell death mechanisms to novel therapeutic opportunities

**DOI:** 10.3389/fimmu.2026.1804503

**Published:** 2026-04-01

**Authors:** Hongyang Chen, Jingsheng Men, Tianyou Gao, Fengli Ma, Zhuangzhuang Jia

**Affiliations:** 1 School of Basic Medical Sciences, Yunnan University of Chinese Medicine, Kunming, Yunnan, China; 2 Yunnan Key Laboratory of Integrated Traditional Chinese and Western Medicine for Chronic Disease in Prevention and Treatment, Kunming, Yunnan, China; 3 Key Laboratory of Microcosmic Syndrome Differentiation, Education Department of Yunnan, Kunming, Yunnan, China

**Keywords:** inflammatory cell death, ischemia-reperfusion injury, PANoptosis, PANoptosome, programmed cell death

## Abstract

Ischemia-reperfusion injury (IRI) can activate multiple cell death pathways, leading to dysfunction of multiple organs. PANoptosis is a new inflammatory cell death form recently discovered and it is also an integrated cell death mode, which includes the features of pyroptosis, apoptosis, necroptosis. Rather than a simple addition of these routes, PANoptosis is a special type of biology process with its own regulatory mechanism and plays an important regulatory role in the occurrence of IRI. The review focuses on the concept of PANoptosis, elaborating on its molecular mechanisms and detection methods, with a particular emphasis on exploring the regulatory networks of PANoptosis in various IRI. In addition, various potential treatment strategies targeting PANoptosis analyzed in depth, including small molecule inhibitors, natural products, gene intervention, and stem cell therapy. The overall goal is to clarify the importance of PANoptosis in the pathological mechanism of IRI and to explore the possibility of using it as a focus for clinical treatment.

## 1 Introduction

Ischemia-reperfusion injury (IRI) represents a clinically significant pathological entity frequently observed in settings including myocardial infarction, ischemic stroke, and solid organ transplantation involving hepatic or renal grafts. IRI not only causes cellular damage due to the interruption of oxygen and nutrient supply during the ischemic phase, but also exacerbates tissue injury due to oxidative stress and inflammatory cascade during the reperfusion phase (1–3). Programmed cell death (PCD) is important to maintain physiological homeostasis (4), which is the core link of IRI, determining the severity of the injury and the prognosis. The traditional researches mainly focused on single death pathways such as apoptosis, necroptosis, and pyroptosis, but therapies that target single PCD pathway have low success rate in alleviating IRI (5, 6). PANoptosis is encouragingly proposed to be a comprehensive cell death paradigm including molecular features of pyroptosis, apoptosis and necroptosis. This coordinated effort is carried out by a multiprotein referred to as the PANoptosome. This model not only integrates the signaling pathways of three classical PCD mechanisms but also reflects their synergistic effects, compensating for the cell death phenomena that could not be fully explained by the previous traditional single models (7, 8). PANoptosis keeps occurring throughout various IRI models. Furthermore, the regulation of key participants in PANoptosis has shown to have a major effect on alleviating tissue damage, making it a potential target for therapy (9–11).

Current evidence increasingly implicates PANoptosis is a key driving factor of IRI in its complex pathological chain. Though still being explored in this research landscape, this research effort on understanding PANoptosis in IRI has the potential to help with how we understand the workings behind IRI and also point to new ways to try to treat such conditions. This review mainly discusses the discovery and concept of PANoptosis, its molecular mechanisms involved, and its effects on IRI in various organs with the intention to create a more detailed understanding of this integrated cell death process. Moreover, potential strategy for targeting PANoptosis for therapy is explored to give a basis for developing more effective treatments for IRI.

## 2 Overview of PANoptosis

### 2.1 Cross-talk among apoptosis, necroptosis, and pyroptosis: discovery and concept of PANoptosis

Common PCD plays a crucial role in regulating cell fate, among which apoptosis is a very common non-inflammatory type of PCD that has been extensively studied (12). It is primarily mediated by two classical pathways: the extrinsic death receptor pathway and the intrinsic mitochondrial pathway, both of which rely on the activation of cysteine-aspartic protease (caspase) family proteases. The extrinsic pathway is triggered when death receptors on the cell membrane, such as Fas and tumor necrosis factor (TNF) receptors, bind to their ligands, stimulating downstream procaspase-8/10, and forming the death-inducing signaling complex (DISC), which subsequently activates downstream effector caspases such as caspase-3, caspase-6, and caspase-7, leading to apoptosis (13). This pathway mainly responds to external signals and is widely involved in immune regulation and cell clearance under pathological conditions. The intrinsic pathway is activated by intracellular stress factors, prompting cytochrome C (CytC) to be released from the mitochondrial membrane into the cytoplasm, forming an apoptosome with apoptotic protease activating factor-1 (Apaf-1) and pro-caspase 9, activating caspase-9 and subsequently activating the effector-type caspase. B-cell lymphoma-2 (Bcl-2) family proteins participate in initiating related processes by modulating the permeability of the mitochondrial membrane (13, 14). The p53 protein can promote apoptosis via the mitochondrial pathway, with some mechanisms involving its interaction with Bcl-2 family proteins, enhancing mitochondrial membrane permeability (15). The two pathways are intertwined, ensuring the precise execution of apoptosis and the determination of cell fate (16). However, when apoptosis is suppressed by diminished caspase-8 activity, cells may enter an inflammatory death mode called necroptosis—unlike traditional apoptosis. This death mode does not rely on caspases, but regulates cell death through complex signaling pathways. Subsequently, under the influence of receptor-interacting protein kinase 1 (RIPK1) and receptor-interacting protein kinase 3 (RIPK3), necrosome formation is triggered, with RIPK3 phosphorylating and activating the mixed lineage kinase domain-like protein (MLKL). Activated MLKL forms oligomers and moves onto the cell membrane, causing membrane rupture and content release, thereby triggering strong inflammatory response (17–19). Pyroptosis is another form of inflammatory cell death, primarily relying on inflammasome activation, characterized by the formation of membrane pores mediated by Gasdermin D (GSDMD), resulting in cell swelling, membrane rupture, and massive release of cellular contents, subsequently provoking a strong inflammatory response (20). The activation of pyroptosis depends on the induction of inflammasomes, predominantly through two mechanisms. The first is the canonical pathway, orchestrated by caspase-1. The second, or non-canonical pathway, is executed by caspase-4/5/11. Additionally, caspase-3 and caspase-8 can also induce pyroptosis, demonstrating its diverse activation mechanisms (21, 22). In the classical pyroptosis pathway, pattern recognition receptors within cells, such as nucleotide-binding oligomerization domain (NOD)-like receptor family pyrin domain containing (NLRP) 3 and absent in melanoma 2 (AIM2), can sense pathogen-associated molecular patterns (PAMPs) and damage-associated molecular patterns (DAMPs), assembling into inflammasomes and activating caspase-1. The activated caspase-1 not only cleaves GSDMD to initiate pyroptosis but also facilitates the conversion of pro-interleukin (IL)-1β and pro-IL-18 into mature pro-inflammatory cytokines, thereby amplifying the inflammatory response (23, 24). The non-classical pathway directly recognizes lipopolysaccharides (LPS) in the cytoplasm through caspase-4/5/11, activating cleaves GSDMD and embedding N-terminus and disrupting the integrity of the cell membrane triggers pyroptosis (25). Furthermore, the regulation of pyroptosis involves various molecules, including different protein cleavage sites and activation mechanisms of Gasdermin family members, with its complexity exhibiting diversity under different pathological conditions.

It was previously believed that these three types of PCD are discrete and independent, with no crossover regulation. However, in 2016, Kuriakose first observed the coexistence of apoptosis, pyroptosis, and necroptosis in macrophages infected with influenza A virus (IAV) originating from mouse bone marrow (26). Subsequently, research has continuously emerged to confirm the complex interactions between apoptosis, pyroptosis, and necroptosis (27). Among them, the caspase family plays a key role in the crosstalk of the three. Caspase-8 is considered a critical connecting molecule between pyroptosis, apoptosis, and necroptosis. Specifically, caspase-8, as a typical initiator caspase, can mediate the classical apoptotic process by activating the downstream effector caspase-3, but its inactivation drives necroptosis (28). Caspase-8 not only participates in apoptosis, but also triggers pyroptosis by regulating inflammatory responses and promoting the formation of inflammasomes (29). In addition, by cleaving RIPK3, caspase-8 acts as a negative regulator of the NLRP3 inflammasome, inhibiting pyroptosis and IL-1β production (30). Secondly, caspase-1, as a typical inflammatory caspase of pyroptosis, mainly induces pyroptosis by cleaving GSDMD to form membrane pores. However, under certain pathological conditions, changes in caspase-1 activity can affect the activation state of caspase-3, thereby altering the form of cell death. For example, in an epilepsy model, after inhibiting GSDMD-mediated pyroptosis, caspase-1-driven caspase-3-mediated apoptosis is activated, leading to massive neuronal death and exacerbation of the condition (31). Other studies have shown that in the absence of GSDMD, bid-induced caspase-3 cleavage can mediate apoptosis (32). In addition to GSDMD, other members of the gasdermin family, such as GSDME, also participate in the cross-regulation between pyroptosis and apoptosis. Caspase-3 can cleave GSDME and generate N-terminal fragments, mediating the transition from apoptosis to pyroptosis (33–35), indicating that there is a dynamic negative feedback and conversion mechanism between pyroptosis and apoptosis.

RIPK1 is regarded as a kind of “branch point” in cell death signal, which can form complex I to promote cell survival, with activating nuclear factor kappa-B (NF-κB) signaling and mitogen-activated protein kinase (MAPK) signaling, while complex II it forms can induce PCD (36, 37). RIPK1 and caspase-8 interact to make the decision of which cell death pathway to follow. Caspase-8 is a trigger of apoptosis, it mediates death receptor-triggered apoptosis and can also cleave RIPK1 and thus directly block necroptosis signaling (38). Also, the self-cleavage of caspase-8 is an important link in its function. In a mouse model of Casp8 self-cleavage site mutation (Casp8^ΔE385/ΔE385^), the cells with the mutated caspase-8 lacking this self-cleavage site showed resistance to Fas-induced apoptosis, however, under the condition of TNF-α-induced, apoptosis could be converted to necroptosis. The cleavage of RIPK1 was significantly reduced, while the complex II and the RIPK1-RIPK3 complex were increased, leading to RIPK3-MLKL dependent necroptosis (39). Beyond necroptosis, RIPK3 also functions as a regulator of pyroptosis, as it can activate NLRP3 inflammasome and promote the secretion of IL-1β when MLKL is insufficient (40), thus inducing the occurrence of pyroptosis. At the same time, oligomerization of MLKL causes K^+^ efflux, and then it activates NLRP3 and causes the release of IL-1β (41, 42), suggesting that RIPK3-MLKL-induced necroptotic signaling could also induces the transformation to pyroptosis. On the other hand, there are also studies showing that when the inflammasome in Lrrk2^G2019S^ macrophages is activated, the formation of mitochondrial GSDMD pores releases mitochondrial reactive oxygen species, thereby inducing the transformation towards RIPK1/RIPK3/MLKL-mediated necroptosis (43).

**Figure 1:**
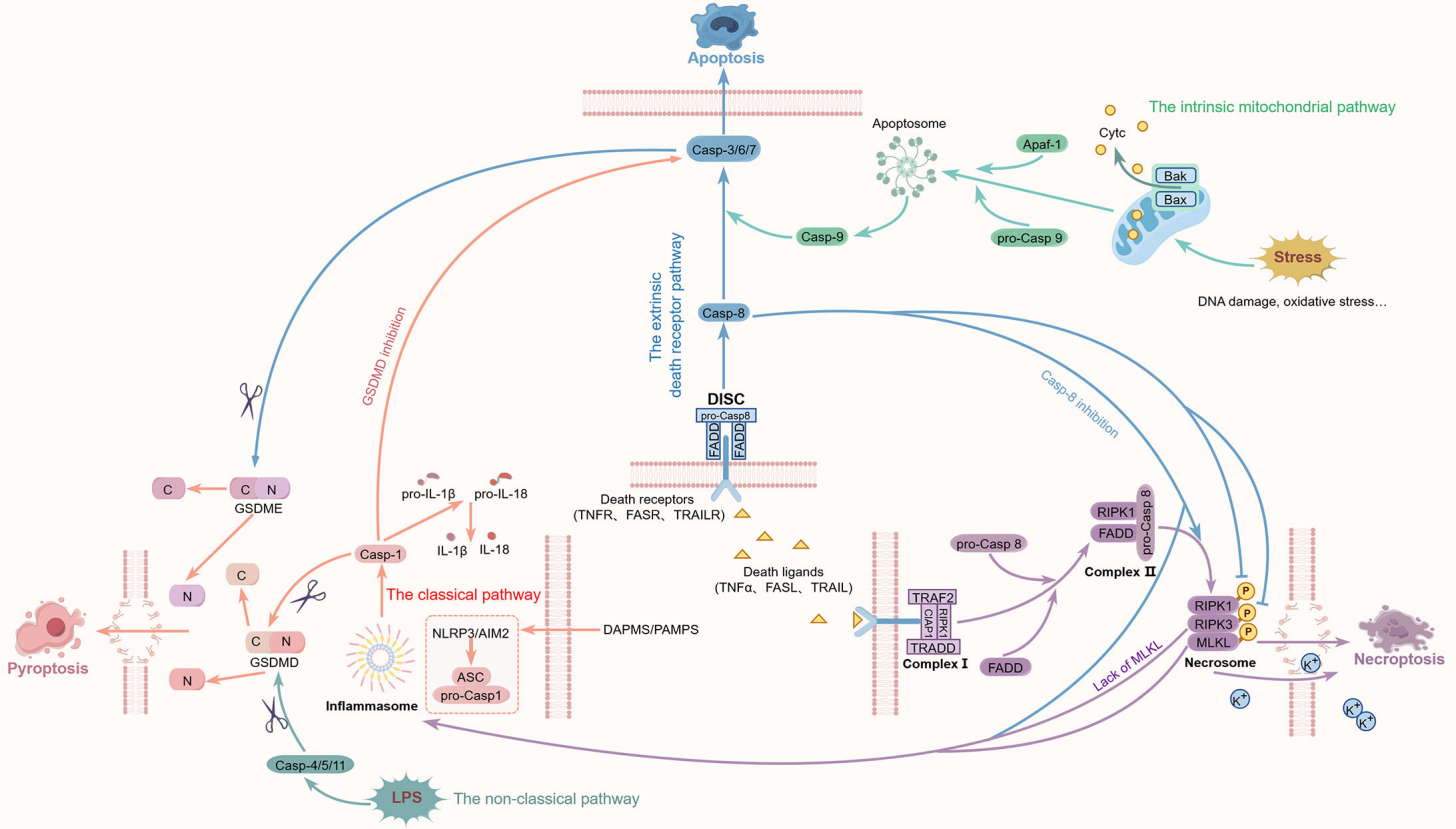
The crosstalk of different pathways during PANoptosis process. Extensive pathway crosstalk underpins the concept of PANoptosis, a unified PCD modality characterized by the simultaneous activation and mutual regulation of pyroptosis, apoptosis and necroptosis.

The above researches confirm that pyroptosis, apoptosis, and necroptosis, although independent signaling pathways, interact with each other at multiple levels. **Figure 1** illustrates the three classic PCD pathways, pyroptosis, apoptosis, and necroptosisas, as well as the crosstalk among them. In 2019, Malireddi first named the crosstalk pattern of cell death involving these three PCD as “PANoptosis” (44). Here, “P”, “A”, and “N” respectively represent pyroptosis, apoptosis, and necroptosis, indicating that PANoptosis shares common characteristics with all three. It is crucial to emphasize that, as mentioned above, the dual and context-dependent functions exhibited by caspase-8 and RIPK1 are not merely examples of simple intermolecular crosstalk but rather form the foundation of the integrated PANoptosis model. Within this framework, they serve as a core decision-making mechanism. The activity states of caspase-8 and RIPK1 constitute a dynamic molecular switch responsible for integrating upstream damage signals and precisely regulating the proportional involvement of apoptosis, necroptosis, and pyroptosis effectors within the unified PANoptosome complex. For instance, when caspase-8 activity predominates, it executes apoptosis by activating caspase-3 while simultaneously inhibiting the necroptosis pathway by cleaving RIPK1/RIPK3, thereby potentially biasing the output of PANoptosis toward an apoptotic phenotype. Conversely, when caspase-8 activity is inhibited or RIPK1 enters a kinase-active state, the necroptosis axis is initiated. The activated necroptosis signal can directly activate the NLRP3 inflammasome, thereby actively recruiting and integrating pyroptosis mechanisms into the same PANoptotic response. Therefore, the “dual roles” of these molecules are essential for the functional plasticity of the PANoptosome. This programmable death output capability fundamentally distinguishes PANoptosis from passive, compensatory pathway redundancy. The essence of PANoptosis lies in the programmed synergistic integration of death pathways, with its core feature being that shared molecular components (such as ZBP1, RIPK1, and caspase-8) can synchronously coordinate the three pathways. Upon specific signal triggers, these pathways are precisely and coordinately activated through the PANoptosome. This integration mechanism, finely regulated by core nodes, transcends the scope of any single death pathway; thus, inhibiting only one mode of cell death is insufficient to block the occurrence of PANoptosis (45, 46).

### 2.2 Molecular mechanism of PANoptosis

**Figure 2:**
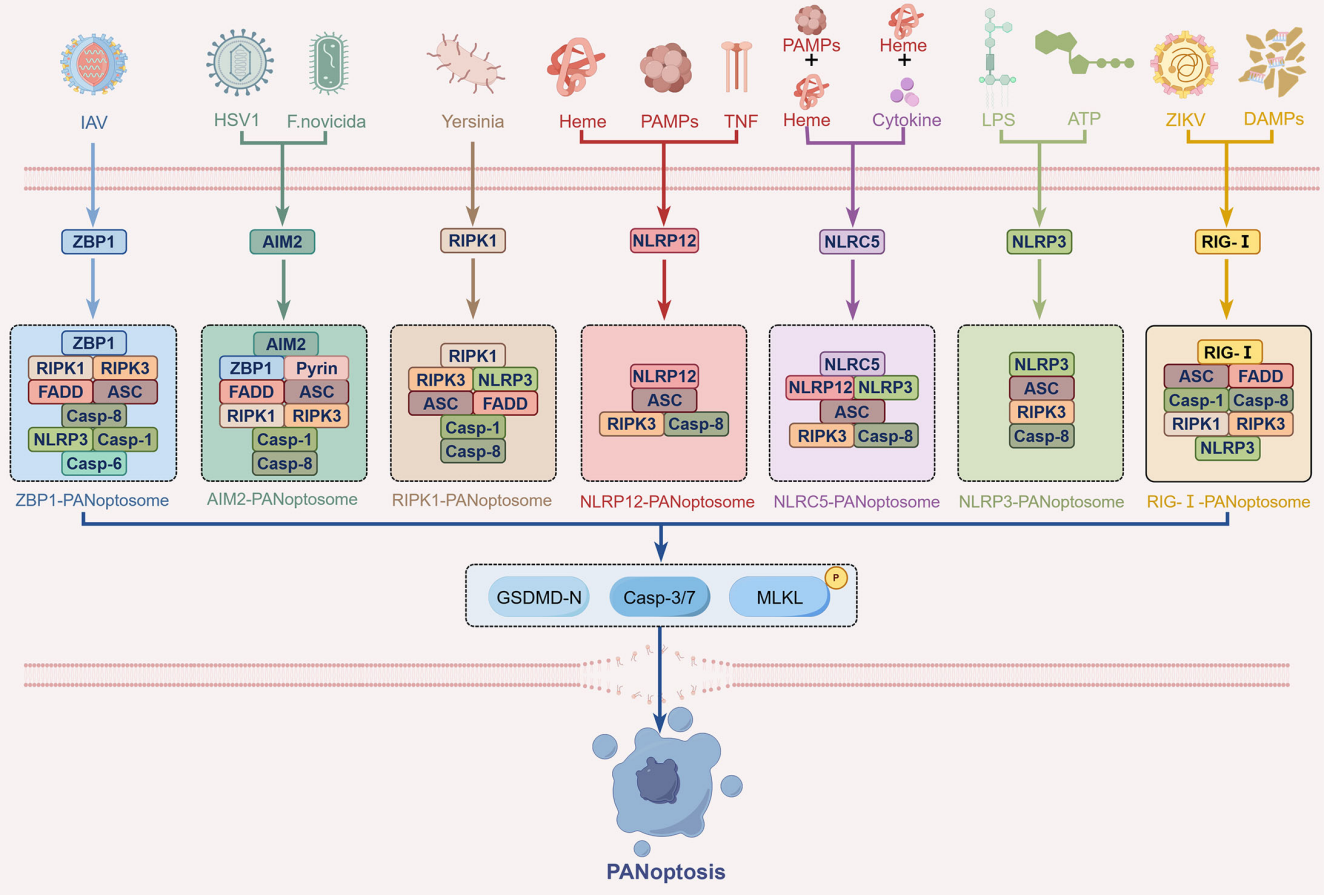
Architecture and assembly of the PANoptosome in PANoptosis. PANoptosis is governed by upstream signals that nucleate the formation of a key polymeric complex, the PANoptosome, which executes the integrated cell death response. Seven types of PANoptosomes with different sensors and regulatory factors have been identified, including the ZBP1-PANoptosome, AIM2-PANoptosome, RIPK1-PANoptosome, NLRP12-PANoptosome, NLRC5-PANoptosome, NLRP3-PANoptosome and RIG-I–PANoptosome.

Like pyroptosis, apoptosis and necroptosis, PANoptosis is also a PCD pathway that is regulated by a series of upstream signals and forms PANoptosome in this process (47), with sensing stimuli such as PAMPs and DAMPs to initiate cell death (7). The structural characteristics of PANoptosome are mainly composed of three core components: sensors, adapters, and effectors (48). Among them, sensors include Z-DNA binding Protein 1 (ZBP1), AIM2, NLRP3, etc., which are primarily responsible for sensing upstream stimuli and transmitting signals downstream (7, 49). Adapters, such as apoptosis-associated speck-like protein containing a CARD (ASC) and Fas associated protein with death domain (FADD), recruit key molecules to form a molecular scaffold, connecting sensors with downstream effector molecules (50, 51), thereby activating effectors to trigger intracellular signal cascades. Effector proteins, including caspase-1, caspase-8, RIPK1, and RIPK3, are the key executioners of cell death and are responsible for performing PANoptosis (47, 52). The composition of PANoptosome can change according to different stimulus factors and the differences in the natural immune receptors that recognize these stimuli (53). Several PANoptosomes have been defined by their distinct combinations of sensors and regulatory factors, with seven major types currently identified, namely: ZBP1-PANoptosome, AIM2-PANoptosome, RIPK1-PANoptosome, NLRP12-PANoptosome, NOD-like receptor family CARD domain containing (NLRC) 5-PANoptosome, NLRP3-PANoptosome, and retinoic acid-inducing gene I (RIG-I)–PANoptosome, with their specific molecular compositions shown in **Figure 2**. In addition, NLRC4 inflammasome has also received considerable attention and may be associated with the occurrence of PANoptosis.

#### 2.2.1 ZBP1-PANoptosome

Upon recognizing Z-shaped RNA produced by viral infection and endogenous nucleic acids, the cytoplasmic nucleic acid sensor ZBP1 triggers the assembly of the ZBP1-PANoptosome. This complex nucleates around ZBP1 and incorporates NLRP3, FADD, ASC, caspases-1/6/8, RIPK1, and RIPK3 (26, 54, 55). In the specific mechanism, ZBP1 relies on its two Z-nucleic acid binding domains at the N-terminus to bind Z-DNA/Z-RNA, and interacts with RIPK1, RIPK3, and other RHIM-containing proteins through its RIP Homotypic Interaction Motif (RHIM) domain (56, 57), subsequently recruiting caspase-8, with promoting MLKL phosphorylation, initiating apoptosis and necroptosis (44). Moreover, ZBP1 activates NLRP3 inflammasome through DISC containing FADD and caspase-8, leading to the maturation and secretion of IL-1β and IL-18, which are necessary during pyroptosis (45). Additionally, caspase-6, as an important component of the ZBP1-PANoptosome, enhances the interaction between RIPK3 and ZBP1 through its non-enzymatic function, facilitating the efficient formation of the PANoptosome and driving the execution of PANoptosis (58).

#### 2.2.2 AIM2–PANoptosome

AIM2 is an important intracellular DNA sensor that activates by recognizing double-stranded DNA (dsDNA) in the cytoplasm, thereby driving the assembly of the AIM2-PANoptosome. The AIM2-PANoptosome was initially discovered during herpes simplex virus 1 (HSV1) and Francisella novicida infections (54). After AIM2 recognizes their dsDNA, it regulates the sensor proteins pyrin and ZBP1, and connects effector enzymes with the help of adaptor proteins ASC and FADD, collectively recruiting RIPK1, RIPK3, and caspase-1, caspase-8 to form the AIM2-PANoptosome (47, 54). Specifically, AIM2 consists of an N-terminal pyrin domain and a C-terminal oligonucleotide-binding HIN domain, with the C-terminal capable of binding dsDNA. When AIM2 recognizes dsDNA, its N-terminal pyrin domain interacts with ASC, promoting inflammasome assembly on one hand, and on the other hand, interacting with ZBP1 to facilitate the formation of the AIM2-PANoptosome (49, 59). In addition, the transcription factor interferon regulatory factor 1 (IRF1) acts as an upstream regulatory factor, promoting the expression and assembly of AIM2 and other PANoptosome components, regulating the occurrence of inflammatory cell death (60), and is expected to provide multi-target interventions for precise regulation of PANoptosis in the future.

#### 2.2.3 RIPK1-PANoptosome

Functioning as a central node within the PANoptosome, RIPK1 orchestrates the convergent death signaling essential for PANoptosis. RIPK1 mediates the assembly of PANoptosomes via its kinase activity, and PANoptosis is triggered. Also, it was found that when infected by Yersinia, the RIPK1-PANoptosome formed and controled the pyroptosis, apoptosis and necroptosis simultaneously. Loss of RIPK1 would block the activation of pyroptosis and apoptosis, but enhance necroptosis (61), indicating that RIPK1 has its own specific function in coordinating different cell death pathways, which also indicates the important role of RIPK1 as a central node in the PANoptosome. Structurally, RIPK1 contains N-terminal kinase domain, C-terminal death domain, intermediate domain that functions as a bridge; and an RHIM domain essential for necroptotic signaling (62). It interacts with the upstream and downstream molecules through these structures to recruit NLRP3, RIPK3, ASC, FADD, and caspase-1/8 to induce PANoptosis (47). The formation of the RIPK1-PANoptosome is controlled by a number of molecules; Transforming growth factor-β-activated kinase 1(TAK1), which is a typical kinase which promotes cell survival, can be blocked by Yersinia, subsequently the host responds to the suppression of TAK1 to assemble the RIPK1-PANoptosome complex to induce PANoptosis (61). So it can be seen that, TAK1 takes on a switch function regarding the regulation of the PANoptosome, inhibiting the phosphorylation of RIPK1 through TAK1 can effectively stop the onset of PANoptosis (55, 63, 64).

#### 2.2.4 NLRP12–PANoptosome

NLRP12 has also been found in recent years to be a key sensor regulating PANoptosis. First, NLRP12 initiates inflammasome assembly by recognizing heme and stimuli such as PAMPs or TNF, activating downstream inflammatory responses. In addition, NLRP12, as a core component of the PANoptosome, coordinates the initiation of various cell death pathways in conjunction with the adapter protein ASC and effector proteins caspase-8 and RIPK3 when the body faces specific microbial invasions, triggering inflammatory necrosis of immune cells (65, 66). This process not only helps to clear infections and damaged cells but may also lead to tissue damage and exacerbation of pathological conditions, exhibiting a dual role. At the molecular mechanism level, the immune regulator IRF1 has been established as a key upstream inducer of both NLRP12 expression and PANoptosome assembly. Toll-like receptors (TLR) 2/4 signaling induces NLRP12 expression through IRF1, further promoting the formation and activation of the PANoptosome (60). In addition, hematopoietic cell kinase is also involved in regulating NLRP12-mediated PANoptosis, binding to the NACHT and PYD domains of NLRP12 to modulate the activity of the PANoptosome, becoming a potential therapeutic target (67).

#### 2.2.5 NLRC5–PANoptosome

As a NOD-like receptor family protein, NLRC5 exhibits untypical caspase activation and recruitment domain (uCARD), the central nucleotide-binding and oligomerization domain (NACHT or NOD), and the C-terminal leucine-rich repeat (LRR) domain. This structural configuration endows NLRC5 with the ability to recognize and bind various PAMPs and DAMPs, thereby participating in the host’s innate immune defense (68, 69). In recent years, it has been confirmed to be closely related to various inflammatory responses and cell death processes. In 2024, Sundaram (70) found that NLRC5 drives PANoptosis under the action of specific ligands (including PAMPs/heme, heme/cytokine combinations), interacting with NLRP12, NLRP3, caspase-8, ASC, and RIPK3 to form a novel cell death complex: NLRC5—PANoptosome, to drive PANoptosis. Notably, while both NLRC5 and NLRP12 can initiate the assembly of PANoptosome, unlike NLRP12, NLRC5 does not regulate the activation of caspase-1 and the release of inflammasome-dependent cytokines, which is the key difference between the two (71).

#### 2.2.6 NLRP3–PANoptosome

NLRP3, as a key sensor in the innate immune system, plays a central role in recognizing various PAMPs and DAMPs signals. It assembles into the NLRP3 inflammasome by recruiting ASC and caspase-1, triggering the maturation and release of intracellular pro-inflammatory cytokines IL-1β and IL-18, inducing pyroptosis (72). Emerging evidence finds that beyond its established role in the classical pyroptosis pathway, NLRP3 is now recognized as an important component of the PANoptosome, where it mediates PANoptosis. Sharma (73) confirmed that NLRP3, as a key sensor, rapidly assembles when cells are stimulated by LPS and adenosine triphosphate (ATP), interacting with adaptor protein ASC and key effector proteins such as caspase-8 and RIPK3 to drive the assembly of the NLRP3—PANoptosome. This assembly process plays a role not only at early time points of classical NLRP3 activation but also shows the formation of PANoptosome and related cell death in cells lacking caspase-1 or GSDMD, indicating that NLRP3 regulates multiple cell death mechanisms in coordination with key molecules from other cell death pathways through the PANoptosome, ensuring the effectiveness of immune defense. Furthermore, the assembly regulation of the NLRP3-PANoptosome involves various upstream signaling pathways and molecules, such as TLR4/NF-κB, thioredoxin interacting protein (TXNIP), and molecules related to metabolic pathways (74, 75). Various small molecule compounds, natural products, and nanomaterials have been found to inhibit PANoptosis by intervening in the assembly or activity of the NLRP3 inflammasome, thereby exerting anti-inflammatory and protective effects (76–78).

#### 2.2.7 RIG-I–PANoptosome

RIG-I is a pattern recognition receptor whose classical function is to recognize viral-derived double-stranded RNA (dsRNA), thereby initiating type I interferon-mediated antiviral immune responses (79). In recent years, the understanding of its functions has continuously expanded, with studies showing that RIG-I can also act as a sensor for DAMPs in non-infectious contexts. Recent advances indicate that under stimulation by specific pathogens or damage signals, RIG-I can serve as an upstream sensing protein that initiates PANoptosome assembly, a mechanism that has been validated in ZIKA virus models and myoglobin-induced pathological models. Activated RIG-I effectively recruits downstream adaptor proteins and effector proteins through its CARD domain or protein interaction interfaces, thereby initiating the assembly of the RIG-I-PANoptosome. In the ZIKV infection model, RIG-I recruits key molecules such as ASC, caspase-1, NLRP3, caspase-8, and RIPK1 (80); whereas in the rhabdomyolysis-related acute kidney injury model, after myoglobin is recognized by RIG-I as a DAMP, it can further aggregate proteins such as ASC, caspase-1, caspase-8, FADD, RIPK1, and RIPK3 (81). These results suggest that RIG-I activation is a common upstream event in different stimulation models, capable of acting as a scaffold molecule to converge components related to pyroptosis, apoptosis, and necroptosis, thereby triggering PANoptosis.

#### 2.2.8 NLRC4–inflammasome

NLRC4 is a sensor of the inflammasome. It is mainly divided into three parts: NACHT domain, CARD domain, and LRR domain. NACHT domain is a Nucleotide binding oligomerization domain, which is mainly responsible for mediating oligomerization and activation of the protein. When the NLRC4 is activated, the NACHT domain drives conformational changes by binding and hydrolyzing ATP, achieving protein self-oligomerization to form a polymeric structure, so as to activate downstream signaling pathways (82). CARD domain takes part in interaction with downstream effector molecules, in particular the binding with ASC adaptor protein and caspase-1, and thus helps the inflammasome to form and become active (83). LRR domain is mainly for recognizing and binding with PAMPs/DAMPs which then activates NLRC4 inflammasome. Although there is no direct evidence linking NLRC4 inflammasome and PANoptosis currently, it is found that NLRC4 may play an important role in PANoptosis (84). NLRC4 detects intracellular PAMPs, notably type III secretion system proteins from Gram-negative bacteria, via NLR family apoptosis inhibitory proteins (NAIP), thereby triggering an immune defense. NAIP binds to NLRC4 by way of a certain “lock and key” kind of activation system, which then promotes the formation and activation of the NAIP-NLRC4 compound (84–86). There are studies showing that *S. Typhimurium* infection activates the NAIP/NLRC4, a process that can culminate in PANoptosis. Therefore, the NAIP-NLRC4 is probably involved in the occurrence of PANoptosis in this process (47, 84), but the correlation between them still needs further evidence to be confirmed.

### 2.3 Detection of PANoptosis

PANoptosis over the last few years has drawn some attention in the pathogenic mechanisms and therapeutics of various diseases. Although constantly brought to light when it comes to its biological relevance, the detection methods for PANoptosis are still in the development stage. The main detection methods currently include the expression detection of key molecular biomarker, the analysis of PANoptosome assembly and observation of cell death morphology, etc. During the execution of PANoptosis, there are a lot of important molecular biomarkers, which can not only serve as transmitters for cell death signals but also detect PANoptosis activity. The biomarkers mainly include NLRP3, members of the caspase family, GSDMD, MLKL, and BCL2-associated X (BAX), etc. Both clinically and in basic research, methods like Western blot, immunohistochemistry and immunofluorescence are used regularly to determine the expression and activation status of these proteins and therefore indirectly find out about the activation of PANoptosis. Furthermore, PANoptosome detection can further confirm the existence of PANoptosis. Currently, we mainly rely on molecular biology technology for the detection of PANoptosome, and co-immunoprecipitation (Co-IP) and immunofluorescence colocalization technology are mainly used. Co-IP technology is good at picking up the interactions between lots of different proteins inside PANoptosome, so it’s an important mean for finding out what the PANoptosome is made of and how it gets together. Take for instance using Co-IP with laser confocal microscopy, it was observed that ZBP1 can interact with key effector molecules such as RIPK3, caspase-8, forming a multi-protein complex that mediates the execution of PANoptosis (87–89). Also, immunofluorescence colocalization technology can make it visible what spatial colocalization state of these molecules within the cell, further confirming the assembly of PANoptosome (90–92).

Following the development of technology, single-cell level PANoptosome detection methods have gradually emerged. Take expansion microscopy in combination with multiplex immunofluorescence staining techniques for example, such methods help us to observe the spatial distribution and dynamic assembly of ASC, caspase-8, and RIPK3 in the PANoptosome at the single-cell level, enriching the understanding of the structure and function of PANoptosome (93). As a composite form of PCD, PANoptosis has complex and diverse morphological manifestations. Therefore, the morphological characteristics and functional assays of cell death are particularly important. The common morphological and functional detection methods include Terminal deoxynucleotidyl transferase dUTP nick end labeling (TUNEL) staining, flow cytometry, and LDH release assays. TUNEL staining is mainly used to detect DNA fragmentation and is an important method for assessing apoptosis; flow cytometry can quantitatively analyze different states of cell death through indicators such as chromosomal damage, membrane integrity, and active enzyme activity; the lactate dehydrogenase (LDH) release assay reflects the rupture of cell membranes and is an important functional indicator for determining cell necrosis or pyroptosis (94–96). Put together, combining these methods helps to comprehensively assess cell death morphology and functional status from multiple perspectives, especially in studying PANoptosis, which can reveal the coexistence and interaction of its multiple death mechanisms.

## 3 PANoptosis in IRI

**Figure 3:**
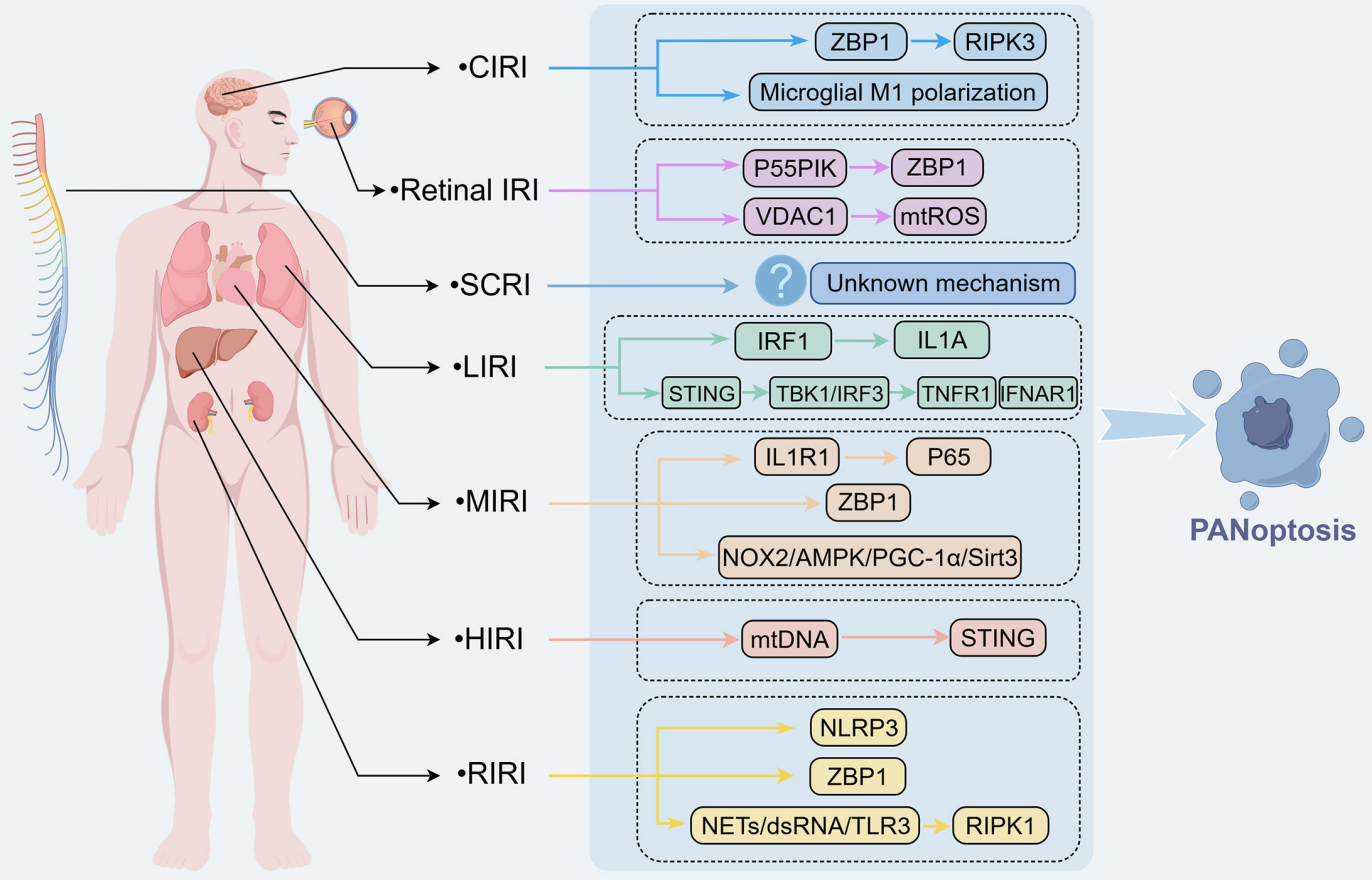
Molecular mechanisms of PANoptosis in organ-specific IRI. PANoptosis is widely present in IRI of multiple organs, mainly including CIRI, MIRI, HIRI, RIRI, LIRI, SCIRI, and Retinal IRI. In these pathological conditions, PANoptosis coordinates cell death responses by regulating downstream key signaling molecules, thereby promoting the progression of cell damage in affected organs associated with IRI.

IRI usually happens after organ transplantation, cardiovascular and cerebrovascular events, and surgeries, it is caused by oxidative stress and inflammation caused by the restoration of blood flow after ischemia, thereby leading to cell death and dysfunction of the organ. The traditional single form of PCD cannot fully explain the complex cell death phenomenon in IRI. As the forms and mechanisms of PCD become increasingly clear, the complexity of cell death in IRI also requires a deeper understanding. Multiple research results indicate that PANoptosis exists in heart, brain, liver, kidney, lung tissue and other tissues, suggesting its central role in IRI. PANoptosis, given its universal occurrence and significant role in pathogenesis of IRI in various organ, this section gives an all round summary about evidence of occurrence of PANoptosis in different types of IRI, and discusses the potential mechanism for its activation and regulation (**Figure 3**, **Table 1**), which will help to develop better intervention strategy for IRI.

**Table 1:** The changes of PANoptosis in different types of IRI.

Types of IRI	Intervention	Assembly of PANoptosome	Key PANoptosis-related genes	Changes in PANoptosis	Reference
CIRI	NA	ZBP1-PANoptosome	ZBP1, RIPK3, MLKL, GSDMD, Caspase-3, Caspase-7	↑	(110)
	NA	ZBP1-PANoptosome	ZBP1, RIPK3, MLKL, GSDMD, Caspase-1, Caspase-3, Caspase-8, ASC, NLRP3	↑	(111)
	CUR-OM-MSCs derived miRNA-423-5p	n.d.	AIM2, ZBP1, Pyrin, Caspase-1, GSDMD, Caspase-3, Caspase-8, RIPK1, RIPK3, MLKL	↓	(112)
MIRI	NA	ZBP1-PANoptosome	ZBP1, RIPK3, Caspase-6, Caspase-8	↑	(10)
	NA	n.d.	NLRP3	↑	(114)
	Gypensapogenin I	n.d.	Caspase-1, Caspase-3, Caspase-8, ASC, NLRP3, GSDMD, RIPK1, RIPK3, MLKL	↓	(115)
HIRI	MtDNA release inhibitor: IMT1B	ZBP1-PANoptosome	ZBP1, GSDMD, Caspase-3, MLKL, RIPK3	↓	(119)
RIRI	NA	RIPK1-PANoptosome	RIPK1, Caspase-1, Caspase-3, MLKL, GSDMD	↑	(11)
	NLRP3 inhibitor: 3,4-methylenedioxy-β-nitrostyrene	n.d.	NLRP3, Caspase-3, GSDMD, MLKL	↓	(125)
	Antioxidant: ultrasmall platinum single-atom enzyme	n.d.	ZBP1, Caspase-1, Caspase-3, Caspase-9, GSDMD, RIPK3, MLKL	↓	(127)
LIRI	STING agonist:diABZI	n.d.	NLRP3, AIM2, Caspase-3, Caspase-8, GSDMD, ZBP1, RIPK3, ASC, MLKL	↑	(130, 131)
	NA	n.d.	Caspase-3, Caspase-8, MLKL, GSDMD	↑	(129)
SCIRI	H_2_S slow-releasing agent: GYY4137	n.d.	Caspase-1, Caspase-3, Caspase-8, NLRP3, MLKL, RIPK1, RIPK3, GSDMD,	↓	(135)
	Melatonin	n.d.	Caspase-1, Caspase-3, Caspase-7, Caspase-8, RIPK1, RIPK3, MLKL, NLRP3, GSDMD	↓	(136)
Retinal IRI	p55PIK synthetic inhibitor: TAT-N24	ZBP1-PANoptosome	ZBP1, RIPK1, RIPK3, Caspase-8, NLRP3, NLRC4	↓	(140)
	Melatonin-NPs	n.d.	Caspase-3, Caspase-7, MLKL, RIPK1, RIPK3, NLRP3, GSDMD, GSDME	↓	(141)
	NA	n.d.	RIPK3, MLKL, NLRP3, GSDMD, Caspase-3, Caspase-8, FADD, ASC	↑	(89)

NA, Not applicable; n.d., Not determined; CUR-OM-MSCs, Curcumin-primed olfactory mucosa-derived mesenchymal stem cells; ↑, Up-regulation; ↓, Down-regulation.

### 3.1 Key integrative factors triggering PANoptosis in IRI

The pathophysiological process of IRI begins with mitochondrial dysfunction induced during the ischemic phase, which serves as the initiating and central link in cellular injury (97). Under ischemic conditions, the activity of the mitochondrial electron transport chain is significantly inhibited, leading to impaired oxidative phosphorylation and a sharp reduction in ATP synthesis (98). Accompanying the energy crisis is the collapse of the mitochondrial membrane potential and abnormal opening of the mitochondrial permeability transition pore (99), resulting in the release of large amounts of pro-apoptotic factors (such as CytC) and mitochondrial DNA (mtDNA). The latter can be recognized as DAMPs by pathways such as AIM2 and cGAS-STING (100–102), providing signals for PANoptosome assembly. Therefore, mitochondrial dysfunction during the ischemic phase and the resulting release of mtDNA constitute a critical bridge linking the initial metabolic crisis to subsequent systemic inflammation and cell death. The “oxidative stress burst” accompanying the reperfusion phase is another core driving factor exacerbating IRI. When blood flow is restored, mitochondrial respiratory chain function has not fully recovered, and increased electron leakage leads to a burst production of large amounts of reactive oxygen species (ROS) (103, 104). The massive ROS, particularly hydrogen peroxide (H_2_O_2_), may indirectly promote PANoptosome activation by oxidatively modifying key signaling proteins or by inducing the release of endogenous ligands (such as oxidized mtDNA) (105–107). Studies have shown that in lower limb IRI models, H_2_O_2_ levels are significantly upregulated during PANoptosis, and inhibiting H_2_O_2_ can alleviate PANoptosis (108). Furthermore, during IRI, calcium overload and endoplasmic reticulum stress are closely intertwined with pathological processes such as mitochondrial dysfunction and oxidative stress, collectively forming a complex stress network. This network converges on activating one or more upstream sensors (such as ZBP1, AIM2, and RIPK1), driving PANoptosome assembly. This integrates various discrete pathological events—including energy metabolism disorders, ion homeostasis imbalance, organelle dysfunction, and oxidative damage—into a unified, synergistically amplified inflammatory cell death program known as PANoptosis, thereby determining the ultimate fate of cells in IRI (109).

### 3.2 Relationship between PANoptosis and IRI in various organs

#### 3.2.1 Cerebral ischemia-reperfusion injury

Cerebral ischemia-reperfusion injury (CIRI) is accompanied by ischemic stroke, which involves multiple pathways of PCD. It is now recognized that in both rodent models and vitro cell models, neurons undergo pyroptosis, apoptosis, and necroptosis simultaneously during the IRI process. ZBP1 is an important regulatory factor of PANoptosis, and is upregulated in the CIRI model. Through using the middle cerebral artery occlusion/reperfusion (MCAO/R) mouse model and the oxygen-glucose deprivation/reoxygenation (OGD/R) cell model, it has been discovered that ZBP1 expression goes up at the same time as other PANoptosis-related proteins, which indicates that ZBP1 might be instrumental in the neuronal PANoptosis caused by CIRI. As ZBP1 is knocked down, the process of PANoptosis is greatly reduced, resulting in less cell damage, reduced area of infarction, and improved neurological functions. On the contrary, overexpression of ZBP1 will aggravate the neuronal damage and the occurrence of PANoptosis, it further confirms the important role of ZBP1-RIPK3 axis on regulating ischemic neuronal PANoptosis (110). This phenomenon has also been validated in endothelial cells, in which the studies also validated that CIRI can induce damaged endothelial cells to assemble ZBP1-PANoptosome, thus causing PANoptosis (111).

In addition, neuroinflammation caused by CIRI is also related to PANoptosis, and the polarization state of microglia is very important for the regulation of this process. It was found through studies that CIRI can induce microglia to undergo a polarization shift to the pro-inflammatory M1 phenotype, promoting PANoptotic cell death of nerve cells. By regulating the polarization state of microglia, especially promoting a switch to the anti-inflammatory M2 type, it is expected to mitigate neuron death resulting from PANoptosis. Olfactory mucosa derived mesenchymal stem cells (MSCs) pretreated with curcumin which secrete miR-423-5p targeting NOD2 and inhibiting NF-κB and MAPK signaling leading to reduced PANoptotic neuron death and improved neurofunctional recovery after CIRI (112). This result not only proves the existence of PANoptosis in CIRI for the first time, but also indicates that regulating microglial polarization to inhibit neuronal PANoptosis indirectly is possible.

#### 3.2.2 Myocardial ischemia–reperfusion injury

Myocardial ischemia-reperfusion injury (MIRI) involves extensive cell death of myocardium cells. From some previous researches we know that if we try to intervene at just one cell death pathway it won’t really work well to reduce MIRI, so we probably need to use some new ways to try to intervene different cell death pathways simultaneously (10). With the continuous deepening of study, the impact of PANoptosis on MIRI has gradually been recognized (7). During MIRI, ZBP1 as a nucleic acid sensor has been found to play an important role in the PANoptosis in myocardial cells. Through recent dynamic transcriptome analysis, it was found that the level of ZBP1 in the MIRI model showed an increasing trend. The expression of various PANoptosis markers in myocardial cells after OGD/R is increased, and the absence of ZBP1 can alleviate the phenomenon, so ZBP1 is an important mediator in PANoptosis of myocardial cells. Mechanically, ZBP1 promotes PANoptosis in cardiomyocytes by promoting the formation of ZBP1/RIPK3/caspase-8/caspase-6 PANoptosome, which eventually leads to the worsening of myocardial damage and myocardial remodeling (10). Moreover, over expression of ZBP1 leads to heart failure, whereas a lack of ZBP1 decreases the area of myocardial infarction, making it a promising candidate as a therapeutic target for MIRI (10, 113).

In addition, it is worth mentioning that inflammatory responses, oxidative stress, and mitochondrial damage are also important to the PANoptosis in MIRI. Interleukin-1 receptor type 1(IL1R1) is a potential related key gene of PANoptosis and its expression greatly increases in myocardial cells under the MIRI circumstance regulated by the MAPK and NF-κB signaling pathways. Machine learning screening and experiment verification all support the role of the IL1R1/p-P65 axis in regulating PANoptosis. Inhibition of IL1R1 can effectively reduce the expression of P65, reduce the inflammatory response and PANoptosis of myocardial cells, and provide new ideas for biomarkers and treatment targets of MIRI (114). Nicotinamide adenine dinucleotide phosphate oxidase 2 (NOX2) will cause the mitochondrial dysfunction and the oxidative injury by inhibiting the AMPK/PGC-1α/Sirt3 signaling pathway, thereby promoting PANoptosis and ferroptosis. The traditional Chinese medicine component compound saponin Gypensapogenin I can downregulate the expression of NOX2, activate the AMPK pathway, maintain the mitochondrial redox state and biogenesis, and significantly inhibit PANoptosis and ferroptosis, reduce the myocardial infarction area, and improve cardiac function, which has a good therapeutic potential (115).

#### 3.2.3 Hepatic ischemia-reperfusion injury

Hepatic ischemia-reperfusion injury (HIRI) usually occurs during operations like liver transplants, liver removals, and other hepatobiliary surgeries, making it harder for patients to recover and causing problems with their transplanted liver function (116–118). The occurrence and development process of HIRI is very complicated, oxidative stress, inflammatory reaction as well as multifarious PCD are involved. In recent years, the regulatory function of PANoptosis in HIRI is becoming more obvious. Among these, the stimulator of interferon genes (STING) pathway serves as an intracellular DNA sensor that is able to recognize the release of mtDNA. Studies have shown that after HIRI, mtDNA leaks into the cytoplasm, and STING signaling pathway is activated, then causing hepatocyte PANoptosis and aggravating liver dysfunction. STING gene KO mice show obvious recovery of liver function, indicating that STING mediated PANoptosis is one of the key causes of the deterioration of HIRI. When treating with IMT1B to inhibit the release of mtDNA, the STING can be inhibited and the PANoptosis can be alleviated to protect the liver function (119). This finding serves as a theoretical basis to for the drug development targeting the STING signaling pathway and is very important for clinic.

Regarding the initiated mechanism of PANoptosis in the liver, the ZBP1 expression level in the liver is rather low in comparison with the brain and heart, which have high ZBP1 expression levels. Correspondingly, AIM2 becomes the primary sensor in the liver, and can trigger the PANoptosis via inflammatory responses. AIM2 activates the inflammasome via the detection of cytosolic DNA, especially mtDNA leakage, thereby causing Inflammatory cell death. Although AIM2 currently has not been founded directly linked to HIRI, the latest studies show that the AIM2-PANoptosome is a key regulator of liver lipid metabolism disorder and a variety of liver diseases, including liver lipid metabolism disorder due to combined exposure of β-Hexachlorocyclohexane and nanoplastics and hepatocyte PANoptosis caused by AIM2-mediated neutrophil extracellular traps (NETs) in acetaminophen overdose-induced acute liver injury (120, 121), which indicates that inhibiting AIM2-PANoptosome may become a target for the treatment of HIRI. Also, we can see from the multi-omics analysis that there are many genes related to PANoptosis and they are all abnormally expressed in the process of HIRI. Through large-scale RNA and single-cell RNA sequencing, it was found that there are many biomarkers associated with PANoptosis in HIRI, including CEBPB, IRF1, HSPA1A, HSPA1B, SERPINE1, and TNFAIP3. Above genes are regulated by NF-κB pathway in HIRI, showing that inflammatory signals have a close link with PANoptosis. Moreover, the PANoptosis process of the liver is related to the inflammatory microenvironment mediated by the MIF and VISFATIN signaling pathways, involving kupffer cells and monocyte-derived macrophages (122). These results contribute to further understand the mechanism of PANoptosis in HIRI, and also give theoretical support for developing new preventions and treatments with targets on these molecules.

#### 3.2.4 Renal ischemia-reperfusion injury

Renal ischemia-reperfusion injury (RIRI) can lead to acute renal dysfunction and increased inflammatory response, and it is also linked to delayed graft function and long-term renal insufficiency following kidney transplantation (123, 124). The role of PANoptosis in renal cell death and inflammatory response has received increasing attention. The latest research shows that, NETs induce RIPK1-dependent PANoptosome assembly in proximal tubular epithelial cells via release of dsRNA, and then trigger PANoptosis which leads to further injury of renal tissue. This process depends on the TLR3 receptors to detect the dsRNA in NETs, and blocking the dsRNA/TLR3 signaling pathway can greatly reduce PANoptosis and renal damage in RIRI, implying that it might be a possible treatment target (11).

As the major protein of the PANoptosome, NLRP3 plays a key role with PANoptosis. Specific inhibitor of NLRP3, 3,4-methylenedioxy-β-nitrostyrene showed obvious protective effect on kidney in RIRI animal model. In animal experiments, the pre-administration of 3,4-methylenedioxy-β-nitrostyrene reduced the tubular injury score, suppressed the expression of caspase-3, GSDMD, MLKL, decreased the apoptosis index, increased the activity of catalase and superoxide dismutase and improved ultrastructure of renal tissue. From these results we can see that blocking NLRP3 specifically is able to inhibit PANoptosis–both pyroptosis, apoptosis, and necroptosis–which is then able to protect the kidney from IRI (125). And also, it is observed that the immune cells like neutrophils, macrophages interact with PANoptosis pathway in RIRI, causing more serious renal tissue injury. Infiltration of immune cells not only can release pro-inflammatory cytokines and activate local inflammatory response but also takes part in the regulation of PANoptosis (123, 126, 127). Signal transmission between immune cells and interaction with renal parenchymal cell, creating a vicious cycle leading to storm of inflammation and storm of cell death, exacerbating the condition of acute kidney injury. And it’s possible to alleviate RIRI by controlling the polarization state of immune cells and stopping them from over-activating. There is research to prove it that antioxidants like Ultrasmall platinum single-atom enzyme, they could not only lessen oxidative stress but also promote the change from M1 to M2 macrophage, slowing down the PANoptosis related inflammatory response and cell death, and thus significantly improving RIRI (127, 128).

#### 3.2.5 Lung ischemia-reperfusion injury

Lung ischemia-reperfusion injury (LIRI) is an essential reason for primary graft dysfunction (PGD), which significantly compromises the long-term outcomes of lung transplant recipients. Its pathological mechanism is very complicated, it is related to oxidative stress, inflammatory response, endothelial barrier destruction, activation of immune cells, and all kinds of PCD pathways (129, 130). Studies about PANoptosis related gene expression on lung transplant patients and animal models show the role of PANoptosis in LIRI after lung transplant. Single-sample gene set enrichment analysis in a human lung transplant model identified a significant upregulation of PANoptosis-related genes during LIRI, including the NLRP3, AIM2 inflammasome, caspase-8, and GSDMD. These key molecules connect PANoptosis to NF-κB and interferon signaling, indicating its role in driving LIRI through inflammatory networks. This process also promotes the occurrence of PGD after lung transplant which indicates regulating those upstream sensors and their activation mechanisms might be some new targets to try to prevent and lower lung injuries and transplanted lung problems (130). While STING pathway has a key part to play in inducing the formation of NLRP3 and AIM2 inflammasome. STING agonists like diABZI will activate STING, and then activate the downstream TBK1/IRF3 signaling pathway, triggering the release of a large number of pro-inflammatory cytokines. Then, these signals activate the TNFR1 and IFNAR1 signaling pathways, further activate ZBP1 and RIPK3/ASC/caspase-8, promote MLKL phosphorylation, trigger necroptosis, and induce apoptosis by caspase-3. In addition, dsDNA that is released intracellularly is recognized by NLRP3 and AIM2, the formation of inflammasomes is induced, leading to the cleavage of GSDMD to form pores, thereby triggering the pyroptosis and the release of mature IL-1β. In essence, this synergistic activation of multiple pathways forms a self-amplifying feedback loop of inflammation and cell death, ultimately inducing widespread inflammation and damage to lung tissue (131). Moreover, researchers have identified cross genes related to LIRI and PANoptosis using bioinformatics and machine learning methods. In animal model of LIRI and peripheral blood mononuclear cells from lung transplantation patients, mRNA and protein expression level of two kinds of PANoptosis related gene, IRF1 and IL1A, were markedly enhanced. Especially for lung transplantation patients with PGD, the expression of IRF1, IL1A is obviously higher than that of non-PGD patients, which may become immune markers of LIRI and also related to the occurrence of PGD (129).

#### 3.2.6 Spinal cord ischemia-reperfusion injury

Spinal cord ischemia-reperfusion injury (SCIRI), this is a kind of serious neurological injury that can be regularly seen after spinal cord operations, often resulting in paralysis and loss of motor functions, which puts a lot of pressure on both patients and society (132–134). PANoptosis has been proved to be playing a key role in nerve damage which happened because of ischemia-reperfusion and it has become an important research direction on the pathogenesis of SCIRI. Some studies have shown the specific manifestations and regulatory mechanisms of PANoptosis in SCIRI. For example, treatment of a rat SCIRI model with hydrogen sulfide (H_2_S) slow-releasing agent GYY4137 can effectively reduce the number of death of spinal cord neurons and improve motor dysfunction. Specifically, the expression of apoptosis-related proteins such as caspase-3, caspase-8, Bax and Bad are downregulated, while Bcl-2 is upregulated which is indicating that H_2_S plays a great role in blocking of the apoptotic pathway. Moreover, the levels of necroptosis related markers p-MLKL, p-RIPK1 and p-RIPK3 are dramatically decreased as well as the cell membrane’s integrity is well retained which shows H_2_S has also necroptosis inhibitory effect. NLRP3 inflammasome related to pyroptosis and the downstream molecules including the cleaved caspase-1 and the cleaved GSDMD expression are also remarkably restricted. The inflammatory response and activation and polarization status of microglia/macrophages are regulated, which decreases the occurrence of neuroinflammation. This shows that H_2_S play a neuroprotective role by inhibiting PANoptosis and excessive neuroinflammation (135).

Another study on melatonin in SCIRI also supports the core role of PANoptosis in this disease. Melatonin, as an endogenous neuroprotective factor, can significantly reverse the activation of neuronal PANoptosis in SCIRI. This result suggests that there was a reduction in nissl bodies damage and an improvement in motor function scores in the melatonin treatment group. The expression of apoptosis markers such as activated caspase-3 is significantly decreased, and the expression of necroptosis and pyroptosis-related proteins is also inhibited. This protective effect is mediated by melatonin receptors, and the use of the receptor antagonist Luzindole can eliminate the neuroprotective effects of melatonin, further confirming that its mechanism is receptor signaling-dependent. In addition, melatonin helps to downregulate the content of IL-1β in microglia, alleviating neuroinflammation, indicating that it protects spinal cord neurons from IRI by inhibiting both PANoptosis and inflammatory responses (136).

#### 3.2.7 Other IRI

Retina and intestine are also important tissues and organs, which also exhibit relevant mechanisms and pathological features of PANoptosis in IRI. Retinal IRI is commonly seen in acute glaucoma and other retinal degenerative diseases (137–139). In terms of retinal IRI induced by acute eye pressure elevation, it is founded that ZBP1-induced PANoptosome, NLRP3 and NLRC4 inflammasomes are all upregulated, triggering PANoptosis.TAT-N24 as a synthetic inhibitor targeting the p55 regulatory subunit of phosphatidylinositol 3-kinase (p55PIK) signaling has been proven to be able to reduce retinal ganglion cell death and retinal structure damage, showing good anti-inflammatory and anti-PANoptosis effect. This also highlights the potential for regulating signaling pathways of PANoptosis for retinal protection (140). And another study developed a ROS responsive and hypoxia responsive nanoparticle system (NPs), loaded with melatonin antioxidant. Melatonin-NPs can reduce hypoxia state and ROS level in the retina and effectively inhibit PANoptosis in retinal ganglion cells and protect retinal neural structure and function and provide new idea for the treatment of acute glaucoma (141). Also, the oligomerization of voltage-dependent anion channel 1(VDAC1) is a major pathologic event regulating PANoptosis in retinal IRI. VDAC1 oligomerization causes mitochondrial dysfunction, which then promotes the formation of the PANoptosome by means of the mitochondrial-derived ROS, and thus PANoptosis. On the contrary, inhibiting VDAC1 oligomerization can greatly reduce the damage of mitochondria and inhibit the occurrence of PANoptosis, so as to reduce the damage and functional loss of retinal cells, indicating that VDAC1 is a possible therapeutic target of retinal IRI (89). Intestinal ischemia-reperfusion injury (IIRI) is an important pathogenic basis for many clinical diseases, such as mesenteric thrombosis, intestinal volvulus, shock, etc (142–144). Though the research specifically regarding PANoptosis and IIRI is very limited currently, taking into account how the intestinal tissue is very sensitive to ischemia-reperfusion, and inflammation and cell death are the core mechanisms underlying the pathological development of IIRI, so PANoptosis would most likely play a part in it. Future research should clarify the regulatory network and actionable targets of PANoptosis in IIRI, in order to provide novel therapeutic strategies for intestinal ischemia-related diseases in the clinic.

## 4 Therapeutic strategies and prospects targeting PANoptosis

**Figure 4:**
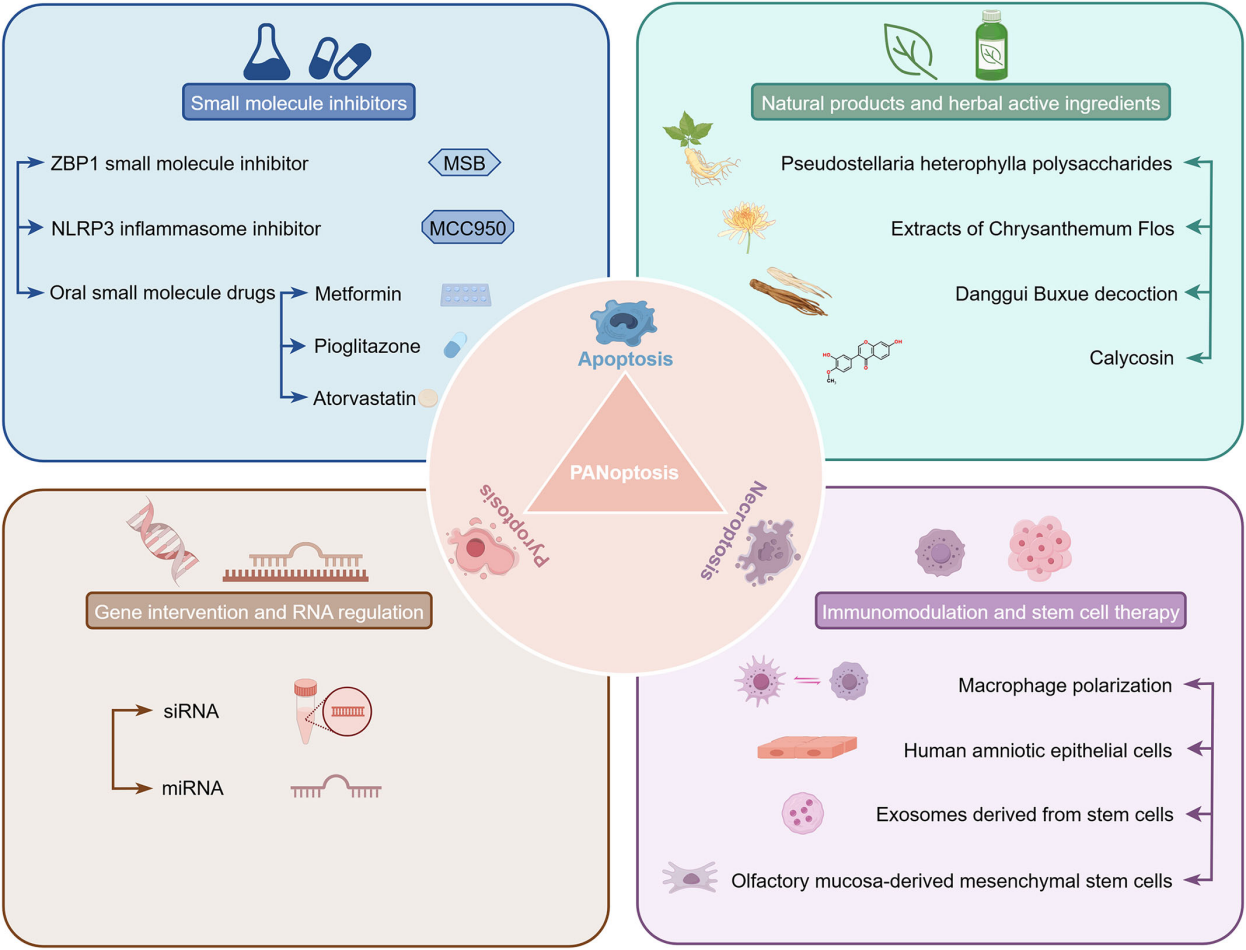
Potential therapeutic strategies targeting PANoptosis for IRI. This figure summarizes key intervention strategies to modulate PANoptosis in IRI, encompassing four principal approaches. These include small molecule inhibitors, natural products and herbal active ingredients, gene intervention and RNA regulation, as well as immunomodulation and stem cell therapy. Each category encompasses several specific sub-strategies.

In therapeutic management of multi-organ IRI, targeting key molecules within the PANoptosis is a hopeful strategic direction, bringing about new alternatives for intervention. Previous studies usually focused only on one type of cell death pattern. Therefore, when attempting to block this pattern, the results were often unsatisfactory, partly because other death paths got activated and did more harm to the tissue (145). Different from that, blocking multiple integrated death process simultaneously via PANoptosis modulation could get rid of such restriction. Though there are no therapeutic agents specifically targeting PANoptosis for IRI which has made it to clinical application yet, but a large amount of preclinical data highlights the potential of this approach. Nowadays, there are some small molecule compounds, including the natural products, especially various herbal ingredients with multiple components and action targets, have shown great potential in regulating cell PANoptosis and alleviating IRI. In addition, new treatment methods like genetic and stem cell therapies also have good application prospects in this field (**Figure 4**). All together give new ways and directions to stop and cure IRI.

### 4.1 Small molecule inhibitor

ZBP1 pharmacological intervention is a prospective treatment for ischemic reperfusion injury (IRI). For example, the novel small molecule inhibitor MSB targeting ZBP1 showed efficacy in reducing IRI both *in vitro* and *in vivo*, and so it could be translated (10). Serving as a nucleic acid sensor, ZBP1 is responsible for activating various PCD signaling pathways during IRI. The decreased expression level of ZBP1 lessens PANoptosis in cardiomyocytes and decreases infarct size. Mechanistically, MSB exerts cardioprotective effects through the high-affinity binding with ZBP1 and inhibition of ZBP1 activation after IRI (10, 146). Apart from suppressing PANoptosis, it seems that MSB might also impact inflammatory reactions and the local environment of the immune system, promoting tissue recovery. At the same time, the NLRP3 inflammasome plays a crucial role as a key participant in pyroptosis and is another important junction of PANoptosis network. By using NLRP3 inhibitors, such as Mcc950, it is possible to inhibit the inflammatory cascade related to PANoptosis and alleviate tissue damage. NLRP3 expression is greatly increased in many organs during IRI. By inhibiting NLRP3 and its mediated inflammatory response, cell death can be suppressed, demonstrating excellent protective effects on the body (147–150). The application strategies of these small molecule inhibitors can provide solutions for regulating the PANoptosis driven by inflammation, and have good application prospects in dealing with IRI problems in the field of neurology and kidney.

Besides the direct-targeting agents mentioned above, certain small molecule drugs that have been used in clinical practice can also modulate PANoptosis in a non-direct manner by modulating metabolism, inflammation and cell death signaling pathways to exert a relieving effect on IRI. For instance, metformin mainly inhibits CIRI by activating the AMPK pathway, which is related to regulating cellular energy metabolism and suppressing inflammatory responses (151, 152). Atorvastatin exerts an indirect regulatory effect on PANoptosis through its antioxidant and anti-inflammatory properties, slowing down of the advancement of ischemic tissue injury (153). Pioglitazone, PPARγ agonist, regulates immune reactions, improves tissue microenvironment and decreases cell death (154). The multi-target mechanism of these drugs provides a good reference for the clinical application, and also indicates that the comprehensive cell death pathways in IRI can be regulated by utilizing existing small molecule drugs.

### 4.2 Natural products and herbal active ingredients

Natural products are getting much attention because they can affect many targets and are safe, so people think they might be good at controlling PANoptosis and making IRI better. Calycosin is one type of naturally occurring isoflavnine, primarily from an herbal medicinal plant *Astragalus membranaceus*, exhibiting multiple biological effects such as anti-apoptosis and inflammation inhibition. In recent times, studies have mainly been centered around its neuroprotective effects and the mechanism of such effects on CIRI especially focusing on how it regulates several PCD pathways such as PANoptosis. In another study carried out via an OGD/R model, simulating CIRI *in vitro*, calycosin was shown to greatly improve the viability of mouse hippocampal neuronal HT22 cells and decrease lactate dehydrogenase release as well as apoptotic rates. Mechanistically, calycosin changed the expression of key genes connected to PANoptosis. It downregulated components of the NLRP3 inflammasome, decreased cleavage of GSDMD and phosphorylation of MLKL, and suppressed RIPK1-mediated necrotic signaling, with the increasing in the Bcl-2/Bax ratio to block the cascade of apoptosis. These coordinated actions did shut down PANoptosis activation and eased neuronal demise. The Molecular docking and dynamics simulations further proved the high affinity and stable binding of calycosin to key targets within the PANoptosis network, which give a structural basis for the mechanism of action. Pharmacokinetics and absorption, distribution, metabolism, excretion, and toxicity (ADMET) analysis were done for calycosin and it was observed that the drug-like property was good and bioactive as well which shows its potential for treatment of ischemic brain injury (155). Calycosin also showed important defensive influence against CIRI brought about by MCAO in rats, which is achieved by inhibiting the neuroinflammation mediated by HMGB1/TLR4/NF-κB axis. Moreover, calycosin also promoted the activity of Nrf2 antioxidant pathway, increased the level of HO-1, NQO1, and reduced oxidative stress-induced neuronal damage (156–158).

Natural products also regulate the expression levels of proteins in the assembly and function of the PANoptosome, thereby modulating PANoptosis. Like extracts of Chrysanthemum Flos, which contain a lot of different flavonoids, have been shown to help with muscle atrophy caused by ischemia. The mechanism is mainly by inhibiting the expression of the PANoptosome related proteins like ZBP1, GSDMD-N, cleaved-caspase-3, p-MLKL, p-RIPK1, and inflammatory cytokine production, promoting muscle protein production and preserve balance of proteins, which show the multifunctional regulatory ability of the herbal active substances for cell death and proteostasis (159). Likewise, in a doxorubicin-induced cardiotoxicity model, Danggui Buxue decoction decreased PANoptosis, and improved cardiac function and tissue integrity by blocking ZBP1-mediated PANoptosome formation. Molecular docking also indicated that active components from the decoction bound to PANoptosis related proteins directly, thus validating that the herbal remedy acted through many targets (160). In addition, polysaccharides derived from *Pseudostellaria heterophylla* were found to down regulate cytochrome p450 enzyme CYP2E1, causing a reduction in mitochondrial damage and inhibit the formation of PANoptosome. These actions together restrained PANoptosis in diabetic-related IRI model and pushed vascular reproduction and tissue renewal (161). In short, these findings strongly show that natural products can not only directly regulate key proteins involved in the assembly process of PANoptosomes, but also regulate related signaling pathways, thereby preventing the occurrence of PANoptosis.

### 4.3 Gene intervention and RNA regulation

RNA interference (RNAi) is a very strong and specific mechanism for silencing genes, which can degrade harmful mRNA transcripts to regulate protein expression, thus determining the course of cells (162–164). It has been found to have distinct advantages in regulating cell death following IRI, providing novel avenues for clinical intervention (112, 165). The core process of RNAi mainly relies on two small molecule non-coding RNAs, small interfering RNA (siRNA) and microRNA (miRNA). By using RNAi technology to target key PANoptosis pathway proteins such as ZBP1 and IL1R1, it has been demonstrated that it can inhibit PANoptosis, downregulate tissue damage and inflammatory response levels, promoting functional recovery of damaged organs. For example, siRNA knockdown of ZBP1 exerts robust cardioprotective effects in MIRI models. ZBP1, as an important part of the PANoptosome, connecting to FADD and RIPK3, promotes the expression of pyroptosis, apoptosis and necroptosis protein in cardiomyocytes to make PANoptosis happen. According to experiments, after using siRNA to knock out ZBP1, the amount of the infarct area is greatly reduced, the histopathological damage is reduced, the levels of cardiac enzymes in the serum of the body are reduced, and the levels of PANoptosis-related proteins are reduced, thus improving the survival of cardiomyocytes (166). *In vitro* results with OGD/R model in H9c2 cells also showed consistent findings. ZBP1 upregulation was associated with more cell death, but blocking it gave protection. Similar to silencing of IL1R1, a receptor involved in inflammatory signaling, which also contributes to the attenuation of PANoptosis in ischemic tissues. The pro-inflammatory effect of IL1R1 is related to the activation of the NF-κB pathway, and blocking the pro-inflammatory effect of IL1R1 through siRNA can prevent the progression of the inflammatory cascade after MIRI, and reduce PANoptosis in cardiomyocytes, alleviating myocardial injury caused by ischemia (114). Additionally, similar protective effects marked by reduced cell death and tissue injury have also been observed in RIRI models post RNAi of relevant genes (167).

There is much attention given to the regulation potential of miRNAs in PANoptosis as a new kind of RNA regulatory mechanism. MiRNAs control PANoptosis through gene targeting, effecting on interconnected events of cell death, inflammation and tissue repair. Taking miR-122 as an example, when HIRI occurs, it enhances the liver’s ischemic tolerance because it controls the PHD1/HIF1α signaling pathway. When miR-122 is overexpressed, it can significantly alleviate liver damage (168). In addition, multiple studies have shown that miRNAs also undergo changes in expression levels in IRI of the heart, brain, and kidneys, providing scientific evidence for the potential of miRNAs in regulating PCD pathways such as apoptosis, necrosis, and autophagy (112, 169, 170). But the specific mechanism and regulatory network of miRNAs in PANoptosis still need more systematic exploration. Based on the various competing endogenous RNA regulatory networks that have been established so far, there are complex interactions among lncRNAs, miRNAs and mRNAs. These interactions play a coordinating and controlling role in multiple PCD pathways, including PANoptosis (171). Future work should focus on identifying important non-coding RNAs, figuring out which genes and signaling pathways they target, and evaluating their therapeutic potential and safety in the field of PANoptosis. It should also be noted that the role of RNAi is not only to knock out genes in cells, but also to deliver siRNA/miRNA to specific sites using engineered vectors, achieving targeted therapy. For example, virus vectors or nanoparticle delivery systems can be used to specifically apply siRNA to inhibit the expression of PANoptosis related genes in animal models such as sepsis, MIRI and RIRI, improving tissue damage and functional defects of related organs (123, 166, 172). The above findings all suggest that RNAi technology can serve as an effective means of precisely regulating PANoptosis, and therefore its potential for clinical application in alleviating IRI cannot be ignored.

### 4.4 Immunomodulation and stem cell therapy

The dynamic changes of immune cells, especially the polarization of cell phenotype and function, have a significant impact on the repair of IRI. The specific polarization state of immune cells can affect inflammation mediated cell death, especially PANoptosis, thereby further regulating tissue damage and recovery. Immunomodulation primarily targets macrophage polarization as of now. Macrophages are very plastic in the IRI microenvironment. The M1 phenotype mostly leads to inflammatory responses by producing lots of cytokines, which worsens the damage to tissues. As opposed to the M1 phenotype, M2 phenotype is linked to inflammation resolution and tissue repair. The PANoptosis is associated with the M1 phenotype transformation, while adjusting to the M2 type state cannot only inhibit the process of inflammatory response, but also suppress key signaling molecules that trigger PANoptosis, reducing the overall cell mortality rate. For example, certain intervention methods that promote M2 phenotype macrophage transformation can inhibit the release of inflammatory factors and promote cell survival in the presence of HIRI and MIRI (128, 173). And some specific non-coding RNAs also become very important regulators of macrophages polarization and PANoptosis, so they might become new kinds of treatment targets (169).

Stem cell therapy, as an emerging immunomodulatory approach, is also a highly promising intervention strategy that can address the issue of IRI. Human amniotic epithelial cells and their derived extracellular vesicles can produce various immune regulating components, which can reduce inflammation to regulate the immune system, preventing cell death and degradation. These combined effects help alleviate IRI in the kidneys and myocardium, thereby helping to maintain tissue structure and function (174, 175). Another study demonstrated that preconditioned olfactory mucosa-derived mesenchymal stem cells (MSCs) regulated the HIF-1α pathway and prevented the occurrence of pyroptosis and apoptosis of microglia after CIRI, indicating that stem cells had the ability of immunomodulation (176). There may be a more optimized solution that combines stem cells with small molecule drugs, which has the potential to produce synergistic defense effects. This strategy makes use of the immunoregulatory properties of stem cells with the focused effects of pharmaceuticals to perform two-pronged intervention into inflammatory and cell death pathways in order to bolster protection and repair of the tissue (177–179). As primary functional vehicles that MSCs secreted, exosomes have become a focus of efforts to develop immunomodulatory strategies. Exosomes can change the immune microenvironment of injured tissues by sending miRNAs, proteins, and other messengers. Previous studies have confirmed that platelet membrane-engineered exosomes of stem cell origin can target and promote the transformation of monocytes into reparative macrophages, thereby achieving tissue repair during IRI by regulating immune function (180). These solid pieces of evidence serve as strong proof that stem cell strategies can effectively regulate PANoptosis in IRI.

## 5 Discussion and future perspectives

With further probing of the pathology mechanisms of IRI, the significance of PANoptosis–an integrated PCD mode–in the process has gradually been revealed. Synthesis of current studies suggests that PANoptosis not only regulates multiple signaling pathways of cell fate but also involves several intersecting signaling pathways and molecular mechanisms, forming a complex network of inflammatory responses, aggravating the tissue damage and functional impairment. The appearance of this cell death mechanism gives us fresh ideas on how to understand the pathological mechanisms of IRI and opened up new directions for clinical practice.

While some progresses have been made on PANoptosis, there are still some limitations, controversies and challenges. On the one hand, although existing studies have reported the simultaneous upregulation of key molecules of pyroptosis, apoptosis, and necroptosis in IRI models, which align with the molecular characteristics of PANoptosis, direct evidence confirming the physical assembly of the PANoptosome complex in the context of IRI remains insufficient, and its formation mechanism requires further elucidation. Currently, many studies have failed to clearly validate the existence of this complex, constituting a common limitation in the literature of this field. On the other hand, in IRI, the precise spatiotemporal dynamic interactions among different death pathways involved in PANoptosis, as well as the specific weight of each death pathway on the outcome of tissue damage, remain key scientific questions that urgently need to be elucidated. Besides, the regulatory mechanisms of PANoptosis show significant differences in different organ IRI. Although cerebral neurons, retinal neurons, renal proximal tubular epithelial cells, and cardiac muscle cells, etc, all exhibit cell death characteristics of PANoptosis, their regulatory molecules and signaling pathways are different. Firstly, the activation of upstream sensors is selective, in CIRI and MIRI, ZBP1 is the core molecule driving PANoptosis; whereas in the liver, its dominant role is replaced by the AIM2 and STING pathways, which primarily respond to cytosolic nucleic acid stress triggered by mtDNA) leakage, thereby initiating the assembly of the AIM2-PANoptosome. Additionally, the core components of the PANoptosome vary across different organ IRI. For example, in CIRI, the ZBP1-PANoptosome predominates, but its specific composition differs subtly between neurons and endothelial cells; in the early stages of RIRI, the RIPK1-dependent PANoptosome dominates, with its assembly closely relying on dsRNA signals derived from NETs. Furthermore, the key signaling initiating PANoptosis exhibit distinct preferences in different organs. In CIRI, MIRI, and retinal IRI, the sensing of intracellular abnormal nucleic acids by ZBP1 constitutes the core initiation mechanism; whereas in HIRI and LIRI, STING recognition of cytosolic DNA is crucial. Notably, mitochondrial dysfunction is not only a downstream outcome of cell death in some organs but also an upstream amplifying signal. VDAC1 oligomerization (retina) and NOX2-mediated oxidative stress (myocardium) further drive PANoptosome assembly by promoting mtROS release. Nevertheless, it must be pointed out that the current research data in this area are still very insufficient, and the identification of the PANoptosome complex in studies is still evolving. These factors somewhat constrain more systematic, in-depth, and accurate comparative analysis of the differences in PANoptosis mechanisms across various organ IRI. As for the application in therapeutics, molecular targeting of PANoptosis has shown some initial protective effects. Inhibitors of the key components of the PANoptosome can effectively decrease the damage of tissues and increase the functional recovery. Still, its translational medicine research is still in its infancy. Mostly studies currently are basic researches which are at an early stage for pre-clinical work, lacking directly targeted clinical trials, and we’ve got a lot more steps before it can be put into practical clinical application, including the specificity of target selection, safety evaluation, and the formulation of personalized treatment strategies. Since PANoptosis involves multiple highly intertwined death signaling pathways, its broad inhibition may interfere with normal immune surveillance, clearance of damaged cells, and tissue repair, thereby potentially increasing the risk of infection, promoting tumorigenesis, or exacerbating side effects such as fibrosis. Further, core molecules of PANoptosis (such as RIPK1, caspase-8) themselves exhibit pleiotropy, participating not only in cell death but also regulating survival, proliferation, and inflammatory signaling pathways; therefore, inhibiting these targets may cause off-target effects, impacting other important physiological functions. It must be recognized that PANoptosis is a defense-damage balance response of the body under pathological conditions. While broad blockade may alleviate acute injury, it could weaken necessary immune clearance capacity, hinder subsequent repair, and even trigger off-target toxicity. This highlights the importance and urgency of shifting current therapeutic strategies from “broad blockade” to “precise balance regulation”.

Looking ahead, in order to achieve deeper progress in the research of PANoptosis in the future, multifaceted efforts are required. Firstly, future research needs to strengthen experimental detection of the assembly and formation of the PANoptosome to directly confirm its core role in PANoptosis, thereby promoting the leap from conceptual description to precise mechanistic understanding of PANoptosis. On this basis, a main focus should be further given to figuring out what exact part PANoptosis plays in different organ tissues and disease stages. This endeavor can not only provide us with a new perspective for understanding pathophysiology mechanisms of IRI, as well as lay the foundation for the implementation of individualized treatment in the future, which is expected to improve therapeutic effects and reduce side effects. Following this line, future researches should further explore the interactions of PANoptosis between different organs, revealing the synergistic or antagonistic effects after different tissue injuries. Secondly, establishing more clinically relevant animal models to mimic the complex and variable ischemia-reperfusion scenarios in clinical practice is also necessary to mechanism study and drug screening. Additionally, given that IRI exhibits distinct stage-specific progression, the microenvironment at different pathological stages (such as hypoxia, oxidative stress, calcium overload, and cytokine storm) shows significant variations, which may lead to dynamic evolution of the dominant cell death pathways. Therefore, future research should integrate the different pathological stages of IRI, systematically combine cutting-edge technologies such as spatial multi-omics, cell type-specific gene editing, *in vivo* real-time dynamic imaging, and high-resolution molecular detection to quantitatively analyze the relative contributions of various cell death modes at different injury stages and delineate the dynamic evolution trajectories of key biomarkers. Only in this way can the dynamic interaction network and spatiotemporal regulatory mechanisms of PANoptosis in IRI be comprehensively revealed, thereby providing a theoretical basis for developing intervention strategies with higher spatiotemporal precision, and ultimately enabling careful balancing of tissue protection benefits and immune homeostasis disruption risks in clinical translation.

In sum, PANoptosis constitutes an integrated regulatory mode of multi-pathway cell death in IRI. Advancing the comprehension of PANoptosis and enhancing its clinical application may facilitate the elucidation of IRI’s complex pathophysiological pathways and provide promising targets for precision treatment. Promoting interdisciplinary collaboration and technological innovation is essential to translate basic research into clinical practice, which may provide a different situation for addressing IRI, ultimately improving patient prognosis and quality of life.
